# Supplementary material for: Genetic Regulation of Transcriptional Variation in Natural Arabidopsis thaliana Accessions
Source: G3 (Bethesda). 2016 May 24;6(8):2319–28. doi: 10.1534/g3.116.030874 (PMC4978887; doi:10.1534/g3.116.030874)
Supplement: Supplemental Material [file supp_6_8_2319__index.html]

Genetic Regulation of Transcriptional Variation in Natural Arabidopsis thaliana Accessions — Supplemental Material 

# Genetic Regulation of Transcriptional Variation in Natural *Arabidopsis thaliana* Accessions

## Supplemental Material for Zan *et al.*, 2016

**Files in this Data Supplement:**

- Figure S1 - Geographical locations of 140 accessions in Schmitz-data. (.jpg, 192 KB)
- Figure S2 - Distance from transcription start site to the top associated eQTL SNP (bp). (.jpg, 224 KB)
- Figure S3 - Illustration of 155 genes with detected cis-eQTLs and no mapped reads. (.jpg, 385 KB)
- Table S1 - 27 genes for which transcripts were only detected in the collection of 107 Swedish *A. thaliana* accessions. (.xlsx, 44 KB)
- Table S2 - 349 cis-eQTL detected in the population of 140 natural *A. thaliana* accessions. (.xlsx, 87 KB)
- Table S3 - 81 cis-eQTL detected in the population of 140 natural *A. thaliana* accessions that were replicated in the population of 107 natural Swedish *A. thaliana* accessions. (.xlsx, 61 KB)
- Table S4 - 2,320 eQTL regulating the expression of 2,240 genes expressed in most of the 140 natural *A. thaliana* accessions. (.xlsx, 259 KB)
- Table S5 - 175 genes affected by eQTL in the population of 140 natural *A. thaliana* accessions for which strong phenotypic effect have already been described in Lloyd et al. (Lloyd and Meinke 2012). (.xlsx, 72 KB)
- Table S6 - 649 cis-eQTL detected in the population of 140 natural *A. thaliana* accessions that were replicated in the population of 107 Swedish natural *A. thaliana* accessions. (.xlsx, 127 KB)
- Table S7 - Tissue specific expression pattern for 111 genes with loss-of-expression cis-QTL and no transcripts in the leaf of *Col-0*. (.xlsx, 43 KB)
