# Supplementary figures and images for: Genetic Regulation of Transcriptional Variation in Natural Arabidopsis thaliana Accessions
Source: G3 (Bethesda). 2016 May 24;6(8):2319–28. doi: 10.1534/g3.116.030874 (PMC4978887; doi:10.1534/g3.116.030874)

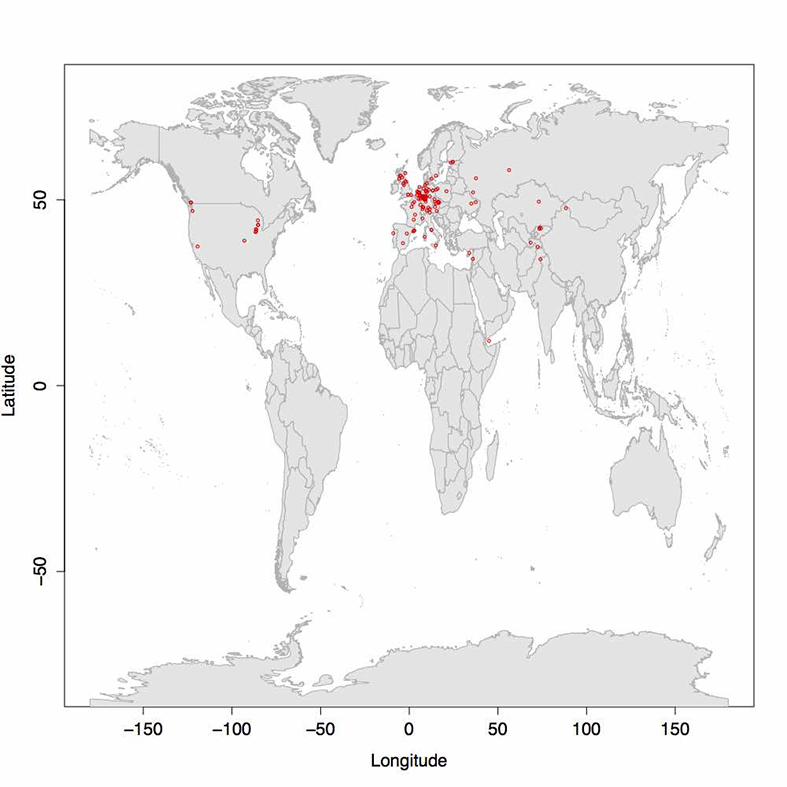

Supplement: Supplemental Material [file supp_g3.116.030874_FigureS1.jpg]

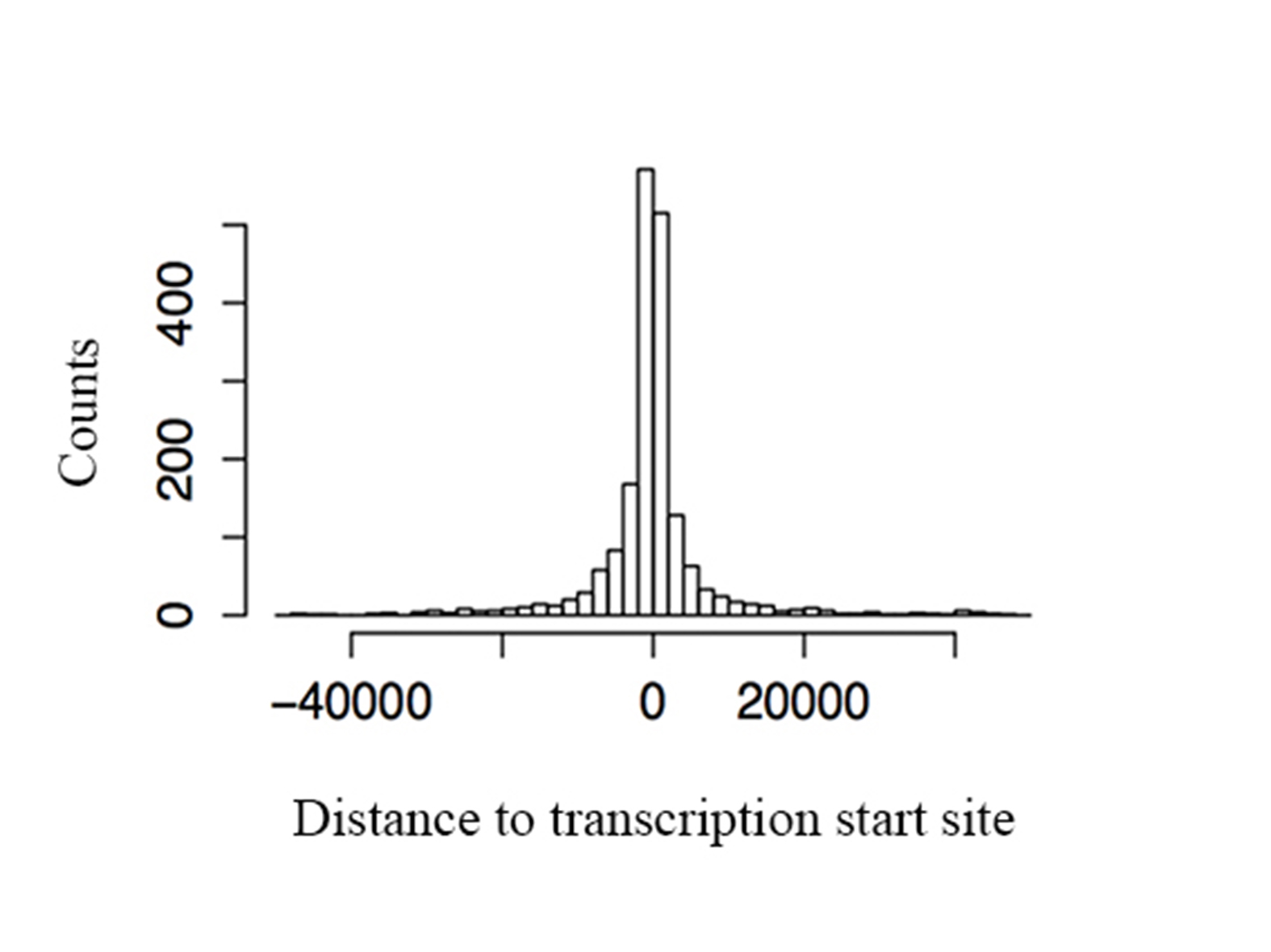

Supplement: Supplemental Material [file supp_g3.116.030874_FigureS2.jpg]

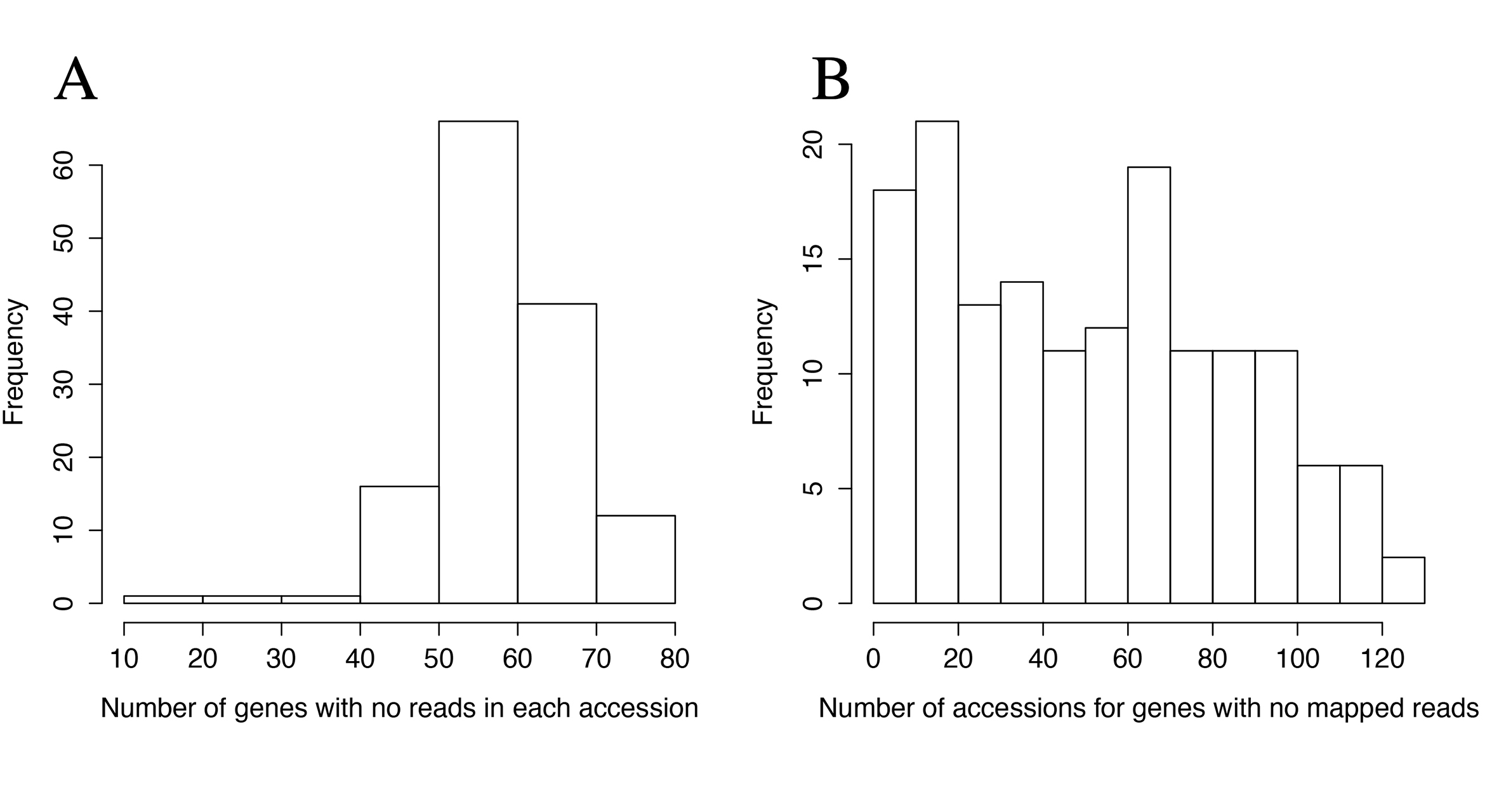

Supplement: Supplemental Material [file supp_g3.116.030874_FigureS3.jpg]
